# Anti-Inflammatory Effect of Three Isolated Compounds of *Physalis alkekengi* var. *franchetii* (PAF) in Lipopolysaccharide-Activated RAW 264.7 Cells

**DOI:** 10.3390/cimb44030094

**Published:** 2022-03-21

**Authors:** Hyun-Jung Park, Hyun Soo Shim, Ah-Reum Han, Eun-Kyoung Seo, Kyu-Ri Kim, Bong Hee Han, Insop Shim

**Affiliations:** 1 Department of Food Science and Biotechnology, Kyonggi University, Suwon 16227, Korea; phj1116@gmail.com; 2 Cell Peprogramming Research Center, Stand Up Science, Seoul 04418, Korea; gustn0930@gmail.com; 3 The Center for Cell Signaling and Drug Discovery Research, College of Pharmacy, Ewha Womans University, Seoul 03760, Korea; hideophj@naver.com (A.-R.H.); phy811@gmail.com (E.-K.S.); 4 Department of East-West Medicine, Graduate School of East-West Medical Science, Kyung Hee University, Seoul 02453, Korea; kyuri_kim@khu.ac.kr; 5 Department of Physiology, College of Medicine, Kyung Hee University, Seoul 02453, Korea; hanbh10@hanmail.net

**Keywords:** *Physalis alkekengi* var. *franchetii*, isophysalin B, physalin, 3′,7-dimethylquercetin

## Abstract

(1) Background: Three isolated compounds from *Physalis alkekengi* var. *franchetii* (PAF) have been investigated to possess a variety of biological activities. Their structures were elucidated by spectroscopic analysis (Ultraviolet (UV), High-resolution electrospray mass spectrometry (HR-ESI-Ms), and their anti-inflammatory effects were evaluated in vitro; (2) Methods: To investigate the mechanisms of action of PAF extracts and their isolated compounds, their anti-inflammatory effects were assessed in RAW 264.7 macrophages stimulated by lipopolysaccharide (LPS). RAW 264.7 cells were treated with different concentrations of *Physalis alkekengi* var. *franchetii* three isolated compounds of PAF for 30 min prior to stimulation with or without LPS for the indicated times. The inflammatory cytokines, interleukin (IL)-1β and tumor necrosis factor (TNF)-α were determined using reverse transcription-polymerase chain (RT-PCR); (3) Results Treatment of RAW 264.7 cells with LPS alone resulted in significant increases in inflammatory cytokine production as compared to the control group (*p* < 0.001). However, with the treatment of isophysalin B 100 μg/mL, there was a significant decrease in the mRNA expression levels of TNF-α in LPS-stimulated raw 264.7 cells (*p* < 0.001). With treatment of physalin 1–100 μg/mL, there was a markedly decrease in the mRNA expression levels of TNF-α in LPS stimulated raw 264.7 (*p* < 0.05). Moreover, TNF-α mRNA (*p* < 0.05) and IL-1β mRNA (*p* < 0.001) mRNA levels were significantly suppressed after treatment with 3′,7-dimethylquercetin in LPS stimulated Raw 264.7 cells; (4) Conclusions: These findings suggest that three isolated compounds from can suppress inflammatory responses in LPS stimulated macrophage.

## 1. Introduction

The fruit calyxes of *Physalis alkekengi* L. var. *franchetii* (PAF) (Mast.) Makino (Solanaceae) have been widely used as traditional and indigenous medicines for therapy for asthma, cough, sore throat, eczema, or urinary problems [1]. There have been phytochemical reports on a variety of types of ingredients from *P. alkekengi* var. *franchetii* (PAF) including physalins, steroids, glycosids, flavonoids, and alkaloids [2]. The extracts and some isolates of this plant have been adopted for the treatment of anti-inflammation, cough, antibacterial [3], antipyretic, antioxidant [3], urinary problem, and antiasthma. The latest pharmacological studies proved its uses in fork medicines, however, the molecular mechanisms of purified compounds remained unclear and were worthy of further exploration. Furthermore, there is little scientific evidence regarding the efficacy of PAF-active compounds in inflammation.

In order to examine their potential anti-inflammatory effects of bioactive compounds from PAF, we isolated three compounds from PAF, identified as 3,7-dimethylquercetin, physalin B, and isophysalin B. The known flavonoid, 3,7-dimethylquercetin was, for the first time, isolated as constituents of PAF and has never been isolated from the genus *Physalis*. The phytochemical and pharmacological activity of three compounds was investigated in the present study.

Based on the highly acclaimed properties of PAF, the present study aimed to evaluate anti-inflammatory (suppression of TNF-α and IL-1β production in LPS-stimulated RAW264.7 cells) activities of ethanol or hexane fraction and their isolated sub-fractions, physalin B, isophysalin B, and 3,7-dimethylquercetin of *P. alkekengi* var. *franchetii* using the RT-PCR.

## 2. Materials and Methods

### 2.1. General Experimental Procedures

Optical rotations were measured on a P-1010 polarimeter (JASCO Co., Ltd, Tokyo, Japan) at 20 °C. HR-ESI mass spectrometric analyses were performed with Waters ACQUITY UPLC system coupled to a Micromass Q-Tof Micro mass spectrometer and Agilent 6220 Accurate-Mass TOF LC/MS system. A YMC Pack Pro C18 column (250 mm × 20 mm i.d., YMC Co., Ltd., Kyoto, Japan) was used for preparative HPLC, along with a Waters system composed of a 1525 binary HPLC pump and a 2487 dual wavelength absorbance detector. Silica gel (230–400 mesh, Merck, Darmstadt, Germany), RP-18 (YMC gel ODS-A, 12 nm, S-150 μm), and Sephadex LH-20 (Amersham Pharmacia Biotech, Amersham, UK) was used for column chromatography. Thin-layer chromatographic (TLC) analysis was performed on Kieselgel 60 F 254 (silica gel, 0.25 mm layer thickness, Merck, Darmstadt, Germany) and RP-18 F 254s (Merck, Darmstadt, Germany) plates, with visualization under UV light (254 and 365 nm) and 10% (*v*/*v*) sulfuric acid spray followed by heating (120 °C, 5 min).

### 2.2. Materials

The fruit calyxes of *P. alkekengi* var. *franchetii* collected in Icheon-si, Kyeonggi-do, Korea were purchased from Chodamchae Co. (Gunpo-si, Kyeonggi-do, Korea), in February 2011 and identified by one of the authors, Prof. Insop Shim (Kyung Hee University, Seoul 130-701, Korea). A voucher specimen (No. EA325) has been deposited at the College of Pharmacy, Ewha Woman’s University. Mouse macrophage RAW 264.7 cells were obtained from the Korean Cell Line Bank (Seoul, Korea). Dulbeco’s Modified Eagle’s Medium; fetal bovine serum (Hyclone; GE Healthcare Life Sciences, Logan, UT, USA); penicillin G (GE Healthcare Life Sciences, Logan, UT, USA); streptomycin (GE Healthcare Life Sciences, Logan, UT, USA); RP-18 F 254s (Merck, Darmstadt, Germany) plates; Kieselgel 60 F 254 (silica gel, 0.25 mm layer thickness, Merck, Darmstadt, Germany); TRIzol reagent (Gibco-BRL Life Technologies; Thermo Fisher Scientific, Inc., Waltham, MA, USA); Taq polymerase (TaKaRa Co., Kusatsu, Shiga, Japan); SYBR green master mix (Invitrogen, Carlsbad, CA, USA).

### 2.3. Extraction and Isolation

The dried fruit calyxes of *P. alkekengi* var. *franchetii* were extracted with 95% methanol (MeOH) overnight at room temperature and then suspended in water and partitioned with hexane, ethyl acetate (EtoAc), and *n*-Butanol (BuOH), sequentially [4]. The fraction was separated by column chromatography (CC). The three physalines (physalin B, isophysalin B, and 3′,7-dimethylquercetin 3β-methoxy-2,3-dihydro-4,7-didehydrophysalin B) were used for anti-inflammation effect. The dried fruit calyxes of *P. alkekengi* var. *franchetii* (8 kg) were extracted with 95% MeOH overnight at room temperature. The MeOH extract (793 g) was then suspended in water, and partitioned with hexane, EtOAc, and *n*-BuOH, sequentially. The hexane fraction (85 g) was subjected to silica gel column chromatography (CC), eluted with gradient mixtures of hexanes-ethanol (1:0→0:1), affording fourteen fractions (EA325H-I–XIV). The fraction EA325H-VIII (1.1 g) was subjected to a silica gel CC eluted with hexanes-acetone (4:1→0:1), yielding 3′,7-dimethylquercetin (rhamnazin) **1** (40.69 mg) and ten sub-fractions (EA325H-VIII-1–10). The fraction EA325H-VIII-7 (14.39 mg) was chromatographed on a Sephadex LH-20 column, using 10 0% MeOH to afford physalin B **2** (1.05 mg). The EtOAc fraction (17 g) was separated by silica gel CC eluted with gradient mixtures of MeOH in CH_2_Cl_2_ (0→1%), affording eighteen sub-fractions (EA325E-I–XVIII). The fraction EA325E-X (1.6 g) was subjected to silica gel CC eluted with gradient mixtures of MeOH in CHCl_3_ (0→1%), yielding physalin B (90.09 mg) and eleventh sub-fractions (EA325E-X-1–11). Sub-fractions EA325E-X-3 (180.3 mg) and EA325E-F10-4 (234.2 mg), respectively, were subjected to reversed-phase CC eluted with gradient mixtures of CH_3_CN-H_2_O (1:1→2:1), and then purified by preparative high-performance column chromatography, using an isocratic mixture of CH_3_CN-H_2_O (2:1, 2 mL/min) as a solvent system to afford isophysalin B (*t*_R_ 68.8 min, 7.14 mg) and 3β-methoxy-2,3-dihydro-4,7-didehydrophysalin B (*t*_R_ 55.2 min 1.10 mg).

**Figure 1:**
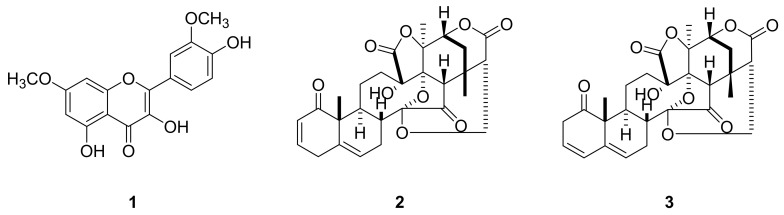
Structures of compounds 1–3 isolated from *P. alkekengi* var. *franchetii*, 3′,7-dimethylquercetin (**1**), physalin B (**2**), and isophysalin B (**3**).

Repeated chromatography of the hexane and ethanol soluble part of the MeOH extract of *P. alkekengi* var. *franchetii* led to the isolation of three known compounds **1**–**3** in Figure 1. Their structure was identified as 3′,7-dimethylquercetin (rhamnazin, **1**),^8^ physalin B (**2**),^9^ and isophysalin B (**3**),^10^ respectively, by analysis of their ^1^H-, ^13^C-, ^1^H,^1^H-COSY, ^1^H,^1^H-NOESY, ^1^H,^13^C-HSQC and ^1^H,^13^C-HMBC spectral data, as well as comparison of their spectroscopic data with those reported previously (Figure 1). Compound **1** was isolated from a plant of the genus *Physalis* for the first time. ^1^H NMR (200 MHz, CDCl_3_) δ ppm: 12.13 (1H, s, OH), 7.92 (d, J = 2.0 Hz, H-2′), 7.85 (1H, dd, J = 8.8 2.0 Hz, H-6′), 7.02 (1H, d, J = 2.0 Hz, H-5′), 6.72 (1H, d, J = 2.0 Hz, H-8), 6.33 (1H, d, J = 2.0 Hz, H-6), 3.94 (3H, s, OCH_3_-3′), 3.93 (3H, s, OCH_3_-7).

^13^C NMR (50 MHz, CDCl_3_) δ ppm: 176.8 (C-4), 166.8 (C-7), 162.0 (C-5), 157.8 (C-8a), 149.9 (C-4′), 148.4 (C-3′), 147.3 (C-2), 137.1 (C-3), 123.6 (C-1′), 123.0 (C-6′), 116.2 (C-5′), 112.2 (C-2′), 104.9 (C-4a), 98.5 (C-6), 92.9 (C-8), 56.5 (OCH_3_-3′, OCH_3_-7).

### 2.4. Cell Culture

The RAW264.7 mouse macrophage cell line has been used extensively to carry out in vitro screens for anti-inflammatory candidate agents [5]. The RAW264.7 cell line response is considered to reflect the potential human de novo response and was used to evaluate the isolated three compounds from PAF for bioactivity and to predict their potential effect in vivo or on primary cells. Mouse macrophage RAW 264.7 cells were obtained from the Korean Cell Line Bank (Seoul, Korea) were routinely kept in a Dulbeco’s Modified Eagle’s Medium supplemented with 10% fetal bovine serum (Hyclone; GE Healthcare Life Sciences, Logan, UT, USA), 100 U/mL penicillin G and 100 µg/mL streptomycin at 37 °C in a 5% CO_2_ air incubator under standard conditions.

### 2.5. LPS-Induced Inflammation and RT-PCR Analysis

RAW 264.7 cells in 6 well plates (1 × 10^5^ cells/well, 500 μL medium/well) were pretreated with various concentrations of *P. alkekengi* var. *franchetii* compounds (1–100 μg/mL) for 4 h prior to incubation for 2 h at 37 °C in an incubator with 5% CO_2_, with or without 1 μg/μL LPS. Total RNA was extracted from the RAW 264.7 cell preparations using 1 mL TRIzol reagent (Gibco-BRL Life Technologies; Thermo Fisher Scientific, Inc., Waltham, MA, USA) and used for cDNA synthesis along with an oligo-dT primer, Moloney murine leukemia virus (MoMLV) reverse transcriptase (200 U), 0.2 mM dNTP, 1.5 mM MgCl_2_, 50 mM KCl, 0.4 M each primer, 0.5 U Taq polymerase (TaKaRa Co., Shiga, Japan) and in a 20 µL final volume of 10 mM Tris-HCl (pH 8.3), in a PTC-100 programmable thermal controller (MJ Research, Waltham, MA, USA). cDNA was synthesized at 25 °C for 5 min and 42 °C for 60 min. PCR was performed with the incubation mixture [2 µL cDNA, 4 µM 5′ and 3′ specific primers. 10× buffer (10 mM Tris-HCl, 0.1% Triton X-100, 250 µM dNTP, pH 8.3, 50 mM KCl, 25 mM MgCl_2_, and 1 U Taq polymerase (TaKaRa Bio Inc., Shiga, Japan)] under the following conditions: 30 s at 94 °C (denaturation), 30 s at 58 °C (annealing), 1 min for extension, and a final extension for 10 min at the end of 25 cycles. The final PCR products were separated with 1.2% agarose gels, stained with ethidium bromide. The band intensities were measured by densitometric analysis ImageMaster TotalLab (Amersham Pharmacia Biotech, Uppsala, Sweden) and were expressed relative to the intensity of the *GAPDH* band.

### 2.6. Quantitative Real-Time PCR (qPCR)

Total RNA was isolated from transiently transfected cells (TRIzol reagent, Invitrogen, CA, USA), reverse transcribed (Superscript III, Invitrogen, CA, USA), and subjected to quantitative PCR analysis using SYBER green master mix (Invitrogen, CA, USA). qPCR was performed with ABI PRISM 7700 Sequence Detection System Instrument and software (Applied Biosystems, Foster City, CA, USA), using the manufacturer’s recommended conditions. The comparative threshold cycle (Ct) method was used to calculate the amplification factor, and the relative number of targets was normalized to GAPDH levels in parallel reactions. The primer sequences are described in Table 1.

### 2.7. Statistical Analysis

The values of the experimental results were expressed as the mean ± S.E.M. Statistical analysis was used with SPSS 25.0 software (SPSS 25 Inc., Chicago, IL, USA). Differences among groups were analyzed using one-way ANOVA and LSD post hoc test. A *p*-value of less than 0.05 was considered statistically significant. Graph generation was followed with Graphpad Prism 6.0 version software.

## 3. Results

### 3.1. Effects of Compounds from Ethanol Extract in Lipopolysaccharide (LPS) Stimulated RAW 264.7 Cells

**Figure 2:**
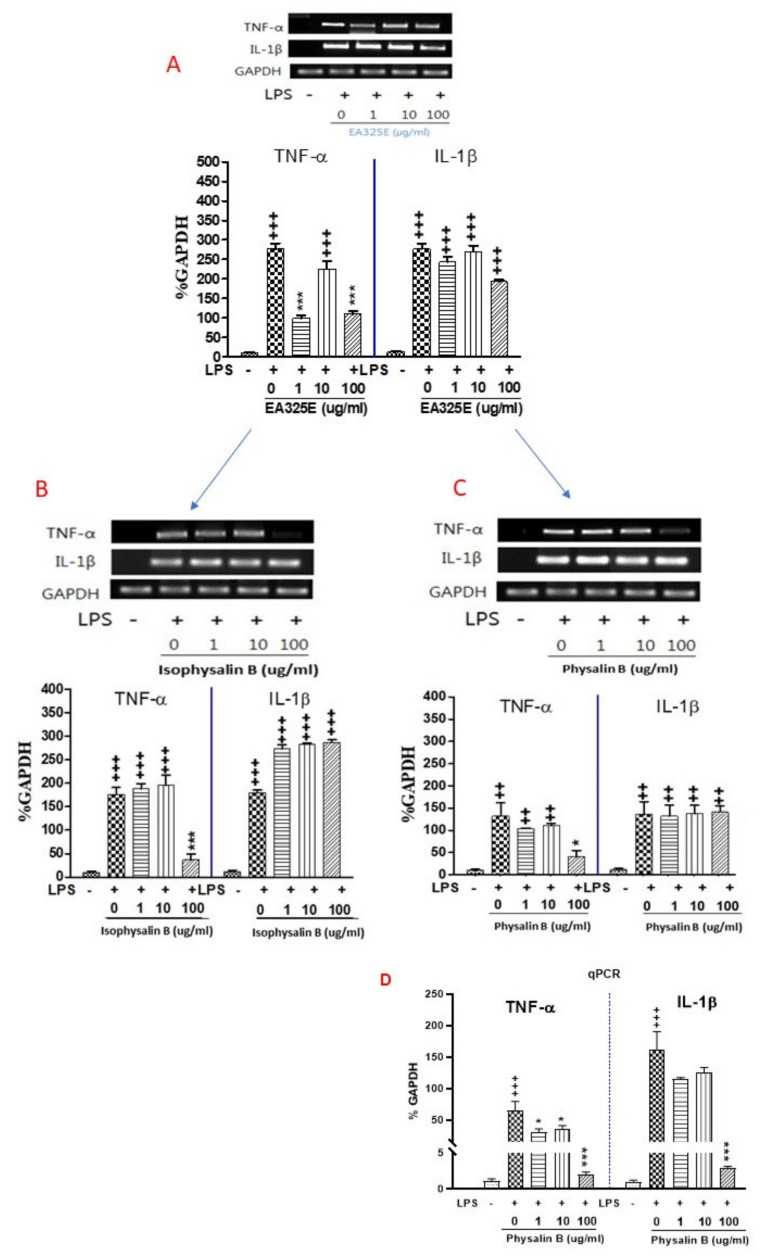
Effects of isolated compounds from ethanol extract in lipopolysaccharide (LPS) stimulated RAW 264.7 cells. Data represent means ± SEM. ^++^ *p* < 0.01, ^+++^ *p* < 0.001 compared to control, * *p* < 0.05, *** *p* < 0.001 compared to LPS. Treatment of RAW 264.7 cells with LPS alone resulted in significant increases in pro-inflammatory cytokines (TNF-α and IL-1β) and mRNA levels as compared to the control group. With EA325E (**A**), Isophysalin B (**B**), and physalin B (**C**) treatment, there was a markedly decrease in the mRNA expression levels of TNF-α in LPS stimulated raw 264.7. (**D**) mRNA expression was determined with q-PCR.

The expression level of TNF-α and IL-1β mRNA in the LPS stimulated RAW 264.7 cells was measured by an RT-PCR (Figure 2A–D). As shown in Figure 2A, treatment with LPS alone of RAW 264.7 cells with LPS alone resulted in significant increases in cytokine production as compared to the control group (*p* < 0.001). However, the expression of TNF-α in the EA325E 100 μg/mL treated group showed a significant decrease as compared to the LPS group (*p* < 0.001).

With physalin 1–100 μg/mL treatment, there was a markedly decrease in the mRNA expression levels of TNF-α in LPS stimulated raw 264.7 (*p* < 0.05). As shown in Figure 2B, treatment of RAW 264.7 cells with LPS alone resulted in significant increases in cytokine production as compared to the control group (*p* < 0.001). However, with isophysalin B 100 μg/mL treatment, there was a significant decrease in the mRNA expression levels of TNF-α in LPS-stimulated raw 264.7 cells (*p* < 0.001). However, there was no significant difference in the mRNA level of IL-1β after treatment of isophysalin B in LPS. As shown in Figure 2C, treatment of RAW 264.7 cells with LPS alone resulted in significant increases in cytokine production as compared to the control group (*p* < 0.001). The expression of TNF-α mRNA levels in physalin B 1–100 μg/mL treated group showed significantly decreased as compared to the LPS group (*p* < 0.05). However, there was no significant difference in the mRNA level of IL-1β after treatment of physalin B among groups. As shown in Figure 2D, treatment of RAW 264.7 cells with LPS alone resulted in significant increases in TNF-α and IL-1β mRNA expression as compared to the control group (*p* < 0.001). However, there was a significant difference in the mRNA level of IL-1β and TNF-α after treatment of physalin B (100 μg/mL) compared to the LPS group (*p* < 0.001).

### 3.2. Effects of Isolated Compounds from Hexane Extract in Lipopolysaccharide (LPS) Stimulated RAW 264.7 Cells

**Figure 3:**
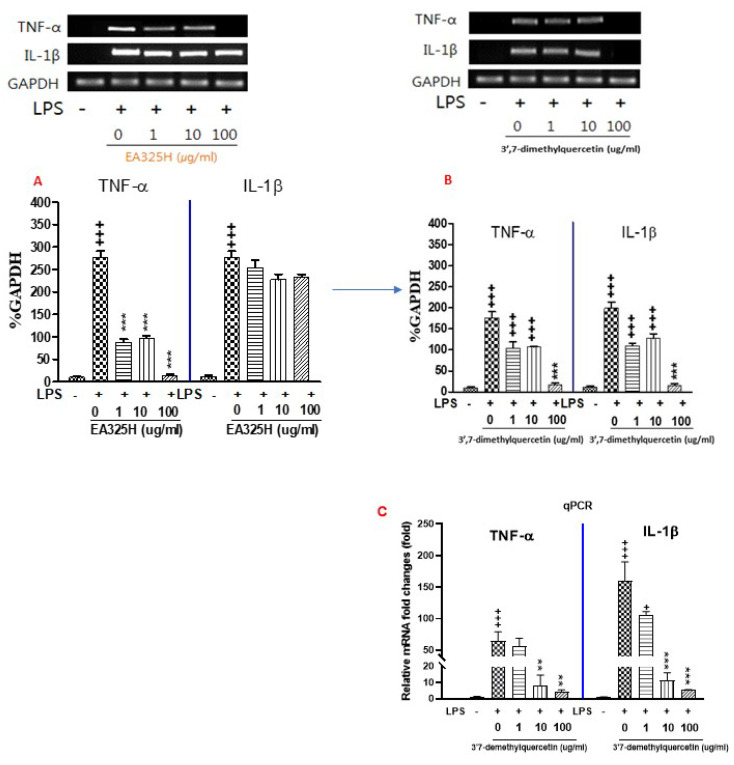
Effects of isolated compounds from hexane extract in lipopolysaccharide (LPS) stimulated RAW 264.7 cells. Data represent means ± SEM. ^+^ *p* < 0.05, ^+++^ *p* < 0.001 compared to control, ** *p* < 0.01, *** *p* < 0.001 compared to LPS. Treatment of RAW 264.7 cells with LPS alone resulted in significant increases in pro-inflammatory cytokines (TNF-α and IL-1β) and mRNA levels as compared to the control group. However, the expression of TNF-α and IL-1β mRNA levels in EA325H (**A**) and 3′,7-dimethylquercetin (**B**) treated group showed a dose-dependent decrease as compared to the LPS group. (**C**) mRNA expression was determined with q-PCR.

EA325H is the hexane fraction, separated from MeOH extracts of *P. alkekengi* var. *franchetii*, and a fraction was subjected to silica gel eluted with hexane–acetone, yielding 3′,7-dimethylquercetin (rhamnazin, Figure 3A,B). The EtOAc fraction, separated by silica gel CC eluted with gradient mixtures of MeOH in CH_2_Cl_2_, was subjected to silica gel CC eluted with gradient mixtures of MeOH in CHCl3, yielding physalin B and isophysalin B. As shown in Figure 3A, treatment of RAW 264.7 cells with LPS alone resulted in significant increases in pro-inflammatory cytokines (TNF-α and IL-1β) and mRNA levels as compared to the control group (*p* < 0.001). The expression of TNF-α in the EA325H 1–100 μg/mL treated group showed significantly decreased as compared to the LPS group (*p* < 0.001).

As shown in Figure 3B, treatment of RAW 264.7 cells with LPS alone resulted in significant increases in pro-inflammatory cytokines (TNF-α and IL-1β) and mRNA levels as compared to the control group (*p* < 0.001). However, the expression of TNF-α mRNA levels in 3′,7-dimethylquercetin 1–100 μg/mL treated group showed a dose-dependent decrease as compared to the LPS group (*p* < 0.05). Also, the expression of IL-1β mRNA levels in 3′,7-dimethylquercetin 1–100 μg/mL treated group showed a significant decrease after 3′,7-dimethylquercetin treatment (*p* < 0.001).

As shown in Figure 3C, treatment of RAW 264.7 cells with LPS alone resulted in significant increases in pro-inflammatory cytokines (TNF-α and IL-1β) mRNA levels as compared to the control group (*p* < 0.001). However, the expression of TNF-α mRNA levels in 3′,7-dimethylquercetin 10 and 100 μg/mL treated group showed a significant decrease after 3′,7-dimethylquercetin treatment (*p* < 0.01). Also, the expression of IL-1β mRNA levels in 3′,7-dimethylquercetin 10–100 μg/mL treated group showed a dose-dependent decrease as compared to the LPS group (*p* < 0.05). These results showed that 3′,7-dimethylquercetin can inhibit LPS-induced inflammation response in RAW264.7 cells.

## 4. Discussion

In this study, we demonstrated the anti-inflammatory effects of three isolated compounds from *Physalis alkekengi* var. *franchetii* on the activation of Raw 264.7 macrophages. The expression of TNF-α mRNA levels in physalin B and isophysalin 1–100 μg/mL treated group was significantly decreased as compared to the LPS group. Importantly, treatment of RAW 264.7 cells with LPS alone resulted in significant increases in pro-inflammatory cytokines (TNF-α and IL-1β) and mRNA levels as compared to the control group However, the expression of TNF-α and IL-1β mRNA levels in 3′,7-dimethylquercetin 1–100 μg/mL treated group dose-dependently decreased as compared to the LPS group. These findings suggest that three isolated compounds from *Physalis alkekengi* var. *franchetii* can strongly suppress the inflammatory response to LPS in macrophages.

Physalins possess an unusual steroidal ring skeleton. Physalins were isolated from physalis species such as physalis angulate, *Physalis alkekengi* var. *franchetii* and *Physalis lancifolia*. Interestingly, these steroids demonstrated diverse pharmacological activities. One of the physalins, physalin B from *Physalis angulata* L. (Solanaceae) is occurring secosteroid with anti-inflammatory activities and antibacterial effects [1,6,7,8,9]. A preclinical study reported that the physalin B inhibits the human HC116 colon cancer cell line viability [10]. Physalin B and physalin F inhibited the growth of several human leukemia cells [11]. Also, isophysalin showed high antibacterial activities against *Escherichia coli* and *Bacillus subtilis* [12]. Another study reported that Physagulin A, physagulin C, and physagulin H could not only inhibit the release of NO, PGE_2_, IL-6, and TNF-α [13].

As shown in Figure 2C, treatment of RAW 264.7 cells with LPS alone resulted in significant increases in cytokine production as compared to the control group. The expression of TNF-α mRNA levels in physalin B 1–100 μg/mL treated group was significantly decreased as compared to the LPS group. However, there was no significant difference in the mRNA level of IL-1β after treatment of physalin B among groups. To further confirm the anti-inflammatory activity of physalin B, the qPCR experiments were performed. As shown in Figure 2D, treatment of RAW 264.7 cells with LPS alone resulted in significant increases in TNF-α and IL-1β mRNA expression as compared to the control group. However, IL-1β mRNA expression was markedly decreased after treatment with physalin B, which were inconsistent results with PCR results. The different results of IL-1β mRNA expression between traditional PCR and qPCR experiments were observed in the present study. The reasons for this difference are not known, but procedural or methodological differences between traditional PCR and qPCR experiments such as primer degradation, contamination, or sample variations might be responsible for these differential results. We observed that there were no significant changes in IL-1β mRNA expression after treatment with physalin B in the PCR experiment. As well known, PCR is relatively a simple qualitative technique and allows only reading the result as presence or absence of expression of IL1β mRNA levels. Even though RT-PCR is a sensitive method for the detection of low-abundance mRNA, there were reported substantial problems associated with its true sensitivity, and specificity inherent in PCR. The observed IL1 β mRNA results from our RT-PCT study may be a non-specific response since this technique compromises the specificity of the reaction. Therefore, it may be unable to distinguish real low-level transcription from false-positive transcription arising from amplification of contaminating genomic DNA with this PCR experiment. In contrast, we found that IL-1β mRNA expression was markedly decreased after treatment with physalin B as seen in Figure 2D in the qPCR as a quantitative technique, providing more reliable and reproducible quantification of IL-1β mRNA. Based on our results of qPCR, we concluded that physalin B at 100 μg/mL completely abolished LPS-induced TNF-α and IL-1β mRNA expression in Raw 264.7 cells, suggesting that the methanol extract of PAF can prevent LPS-induced inflammatory response through the downregulation of IL-1β and TNF-α in RAW 264.7 macrophages. Therefore, it could be possible to consider PAF as a potential therapeutic agent.

Our data also proved the treatment of physalin B and isophysalin strongly suppressed LPS-induced TNF-α mRNA expression. However, relatively few studies have investigated the pharmacological activity of 3′,7-dimethylquercetin. The present study demonstrated that 3′,7-dimethylquercetin inhibited LPS-induced TNF-α and IL-1β mRNA expression in the Raw 264.7 cells. Few studies obtained bioactive 3′,7-dimethylquercetin from citrus wax [14], Siegesbeckia pubecens, [15], and leaves of Marcaranga triloba [16]. Jang et al. reported that 3′,7-dimethylquercetin inhibits cyclooxygenases-1 and -2 by measuring prostaglandin 2 production in cultured Hepa 1c1c7 mouse hepatoma cells [16]. Collectively, these findings suggest that 3′,7-dimethylquercetin has significant anti-inflammatory effects via the regulation of pro-inflammatory cytokine expression.

## 5. Conclusions

In this study, for the first time, our results demonstrated the anti-inflammatory effects of three isolated compounds from *Physalis alkekengi* var. *franchetii* on the activation of Raw 264.7 macrophages. The expression of TNF-α mRNA levels in physalin B and isophysalin B 1–100 μg/mL treated group was significantly decreased as compared to the LPS group. Importantly, treatment of RAW 264.7 cells with LPS alone resulted in significant increases in pro-inflammatory cytokines (TNF-α and IL-1β) and mRNA levels as compared to the control group. However, the expression of TNF-α and IL-1β mRNA levels in 3′,7-dimethylquercetin 1–100 μg/mL treated group dose-dependently decreased as compared to the LPS group. These findings suggest that three isolated compounds from *Physalis alkekengi* var. *franchetii* can strongly suppress the inflammatory response to LPS in macrophages.

## Figures and Tables

**Table 1:** Primer sequences used for the qRT-PCR.

Name	Primer Sequences
Gapdh (mouse form)	F: 5′-ACA CAT TGG GGG TAG GAA CA-3′ R: 5′-AAC TTT GGC ATT GTG GAA GG-3′
TNF-α (mouse form)	F: 5′-GCAGAAGAGGCACTCCCCCA-3′ R: 5′-GAT CCA TGC CGT TGG CCA GG-3′
IL-1β (mouse form)	F: 5′-GGC TGT GGA GAA GCT GTG GC-3′ R: 5′-GGG TGG GTG TGC CGT CTT TC-3′

## Data Availability

Not applicable.
